# Maternal Factors Influencing the Nutritional Status of HIV-Exposed Infants: A 12-Month Follow-Up in Mathare and Kibera Informal Settlements in Nairobi County

**DOI:** 10.1007/s10461-025-04648-4

**Published:** 2025-02-14

**Authors:** Elizabeth Mueke Kiilu, Simon Karanja, Gideon Kikuvi, Linet Muthoki

**Affiliations:** 1 Department of Public Health, University of Bradford, Bradford, UK; 2 Department of Public Health, Jomo Kenyatta University of Agriculture and Technology, Nairobi, Kenya; 3 Department of Public Health, Mount Kenya University, Kiambu, Kenya

**Keywords:** Infant, HIV exposed infant, Infant nutrition, Maternal HIV infection

## Abstract

Optimal infant nutrition is crucial for good health and survival. HIV-exposed infants have a greater incidence of low birthweight than HIV-unexposed infants, predisposing them to malnutrition and a greater risk of HIV infection. A 12-month longitudinal study was conducted on 166 HIV-exposed infants, assessing nutritional status at 6 weeks, 6 months, and 12 months. Fisher’s test and logistic regression analysed the data using WHO growth standards. Ethical approval was obtained (KEMRI/SERU/CPHR/002/3525). Results: *Wasting*: Younger maternal age (18–24 years) presented higher odds of infant wasting across all timepoints: 6 weeks aOR 4.31 (CI: 1.11, 1.83), 6 months aOR 4.49 (CI: 1.09, 27.34), and 12 months aOR 5.49 (CI: 1.41, 32.97). *Stunting*: At 6 months, infants of underweight mothers and those on second-line antiretroviral therapy (ART) regimens had higher odds of stunting aOR 4.76 (CI: 1.36, 16.65) and aOR 5.49 (CI: 1.64, 18.38), respectively. At 12 months, poor maternal ART adherence aOR 4.11 (CI: 1.14, 14.82) and mothers on second-line ART regimens aOR 3.68 (CI: 1.09, 12.49) had increased odds of infant stunting. *Underweight*: At 6 weeks, high maternal viral load aOR 6.33 (CI: 2.31, 17.36) was associated with higher odds underweight infants, whereas employed mothers had lower odds of underweight infants at 6 and 12 months aOR 0.10 (CI: 0.03, 0.32) and aOR 0.22 (CI: 0.09, 0.59) respectively. The results highlight maternal nutrition and ART adherence’s influence on infant nutritional status and HIV vertical transmission risk. The study recommended integrating comprehensive nutritional care into HIV policies and enhancing ART counselling to reduce vertical transmission risk and poor infant growth.

## Introduction

Adequate nutrition in the first two years of life is crucial for good health, growth and development. Informal settlements are strongly associated with inadequate food [1] and other forms of deprivation (assets and subjective poverty) and elevated morbidity and mortality rates [2]. HIV-exposed infants in this environment are further compounded with a double burden of malnutrition and infections. Nutrition in HIV-exposed infants is complex, as it is compounded by several risk factors, such as lower birth weight than that in HIV-unexposed infants and reduced maternal passive immunity, which, in turn, may increase the risk of infections and subsequent poor growth in HIV-exposed infants [3].

Stunting is associated with poor sanitation, repeated exposure to adverse economic conditions, and the interactive effects of suboptimal intake and infections [4]. Wasting in children is a life-threatening result of inadequate intake and/or disease, which results in weakened immunity, developmental delays, and an increased risk of mortality [5]. Underweight status indicates a history of nutritional insult or poor health, whereas wasting status is associated with recent illness and failure to gain weight or weight loss [6]. Despite various forms of malnutrition, the path to management is the same and includes adequate maternal nutrition during pregnancy and lactation, exclusive breastfeeding (EBF) in the first 6 months of life, optimal breastfeeding in the first two years of life, nutritious, diverse, and safe foods in early childhood, and a healthy environment, including adequate sanitation, access to safe drinking water, and proper hygiene [5]. These simple yet effective interventions will lead to a world free of childhood malnutrition, but effective and sustained multisectoral nutrition programming is paramount to see these initiatives put in place.

Malnutrition estimates [7] revealed that Asia and Africa bear the greatest burden of all forms of malnutrition globally. In 2018, more than half (55%) of all stunted children aged less than 5 years lived in Asia, whereas 39% of all stunted children lived in Africa. In Kenya, 26% of children under the age of five were stunted in 2017, exceeding the expected average of 25% for developing countries. Additionally, 4% of children under five were wasted, a rate that was lower than developing countries average of 8.9% [7].

Several factors influence the nutritional status of infants born to mothers living with HIV. This study explored maternal factors that influence the nutritional outcomes of HIV-exposed infants. Maternal HIV-positive status and poor nutritional status reduce passive immunity, which may increase the risk of infection and subsequent poor growth in HIV-exposed and infected infants [8, 9]. Women with a more advanced HIV illness or who have just become infected recently  (i.e. less than two years) are more likely to transmit the virus [10]. Furthermore, appropriate antiretroviral therapy (ART) interventions for either mothers living with HIV or HIV-exposed infants can significantly reduce the risk of postnatal transmission. Shorter durations of breastfeeding are associated with decreases in weight, height, and development [8]. However, in many resource-limited settings, infants who do not breastfeed are up to six times more likely to die from malnutrition, pneumonia, and diarrheal diseases. The dilemma in settings where child mortality is due to these conditions is relatively common, such as sub-Saharan Africa, the strategy has been to balance the risk of infants being infected with HIV postnatally through breastfeeding with the risk of infants dying from other causes if they are not breastfed [11].

## Methods

The study was a prospective cohort study whereby 166 (after adjusting for 40% loss to follow-up) mother‒infant pairs were recruited into the study and followed up for 12 months. Nutritional parameters were measured at three time points: 6 weeks, 6 months (26.12 weeks) and 12 months (52.24). These are the timepoints that HIV exposed infants are assessed for HIV status, hence these timepoints were also used to capture the nutritional parameters. The parameters measured were infant weight for height (measuring wasting), infant weight for age (measuring underweight), and infant height for age (measuring stunting). The corresponding Z scores for these three nutritional categories were recorded and reported accordingly. WHO growth standards and the corresponding Z scores were used, whereby stunting (length for age <− 2 SD), wasting (weight for length <− 2 SD), and underweight (weight for age <− 2 SD) were the cut-off values used.

The study was conducted in 3 public healthcare facilities, namely, the Kibera South Health Centre, Mathare North Health Centre and Mbagathi County Hospital, which serve the informal settlements of Kibera and Mathere. The study population was infants born to mothers living with HIV (who were two weeks old up to ≤ six weeks of age) and enrolled for Early Infant Diagnosis (EID) services. They were selected using simple random sampling technique, with the postnatal register used as the sampling frame.

Nutritional information was obtained from the mothers via a semi-structured interviewer-administered questionnaire and a data abstraction tool. This study used simple rapid assessment techniques aided by WHO indicators to assess feeding practices [12]. The reliability of the data collection tool was tested via Cronbach’s alpha, whereby the reliability indices obtained in the study were all above 0.7 and deemed acceptable.

Data cleaning, coding, and analysis were performed via STATA Version 14. Descriptive statistics were used to explore and summarize the data. Infant nutritional status and Fisher’s exact test at the univariable level and logistic regression [13] at the multivariable level were used. The study was powered at 80%. All significant variables at p= < 0.1 were used in the forward and backwards modelling process via the Bayesian Information Criterion (BIC).

Ethical approval was obtained from the Kenya Medical Research Institute (KEMRI) Scientific Ethics Review Unit (SERU), KEMRI/SERU/CPHR/002/3525. Informed written consent was obtained from all the participants, and the benefits and risks of the study were also explained to them. Confidentiality, privacy, and anonymity were maintained, and participants were free to withdraw from the study without implications for the mother–infant pairs.

## Results

### Nutritional Status of Infants Enrolled in EID Services for HIV

The nutritional status of the infants was assessed at three time points: 6 weeks, 6 months (26.12 weeks), and 12 months (52.24 weeks). The WHO growth standards and the corresponding Z scores were used, whereby stunting (length for age < − 2 SD), wasting (weight for length < − 2 SD), and underweight (weight for age < − 2 SD) were the cut-off values used.

**Fig. 1:**
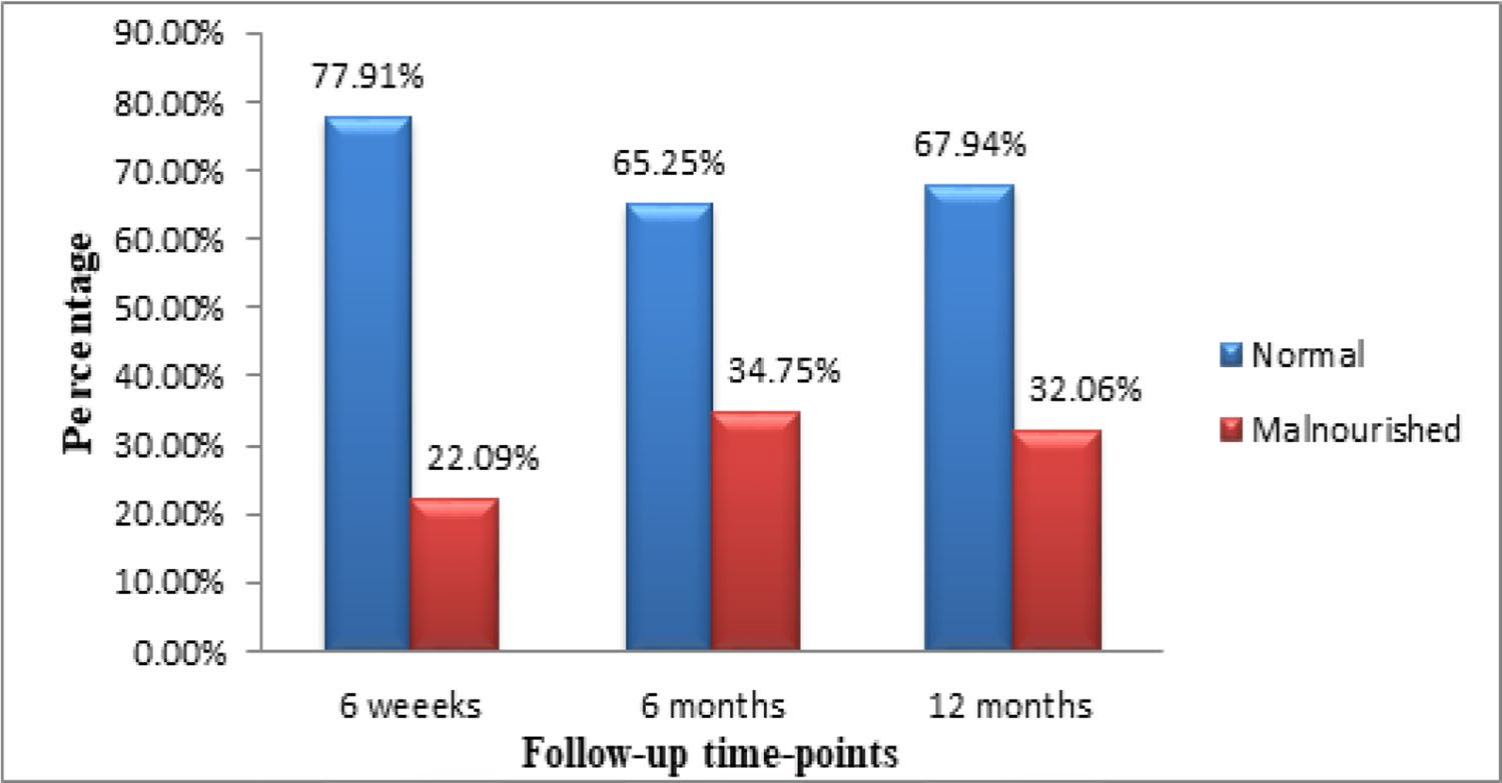
Overall infant nutritional status over 12 months of follow-up

The overall infant nutritional status over the 12-month follow-up period was as follows: at 6 weeks, 36 (22.0%) of the infants were malnourished, with the highest degree of malnutrition being experienced at 6 months 48 (34.75%). At 12 months, 42 (32.06%) of the infants were malnourished (Fig. 1).

**Fig. 2:**
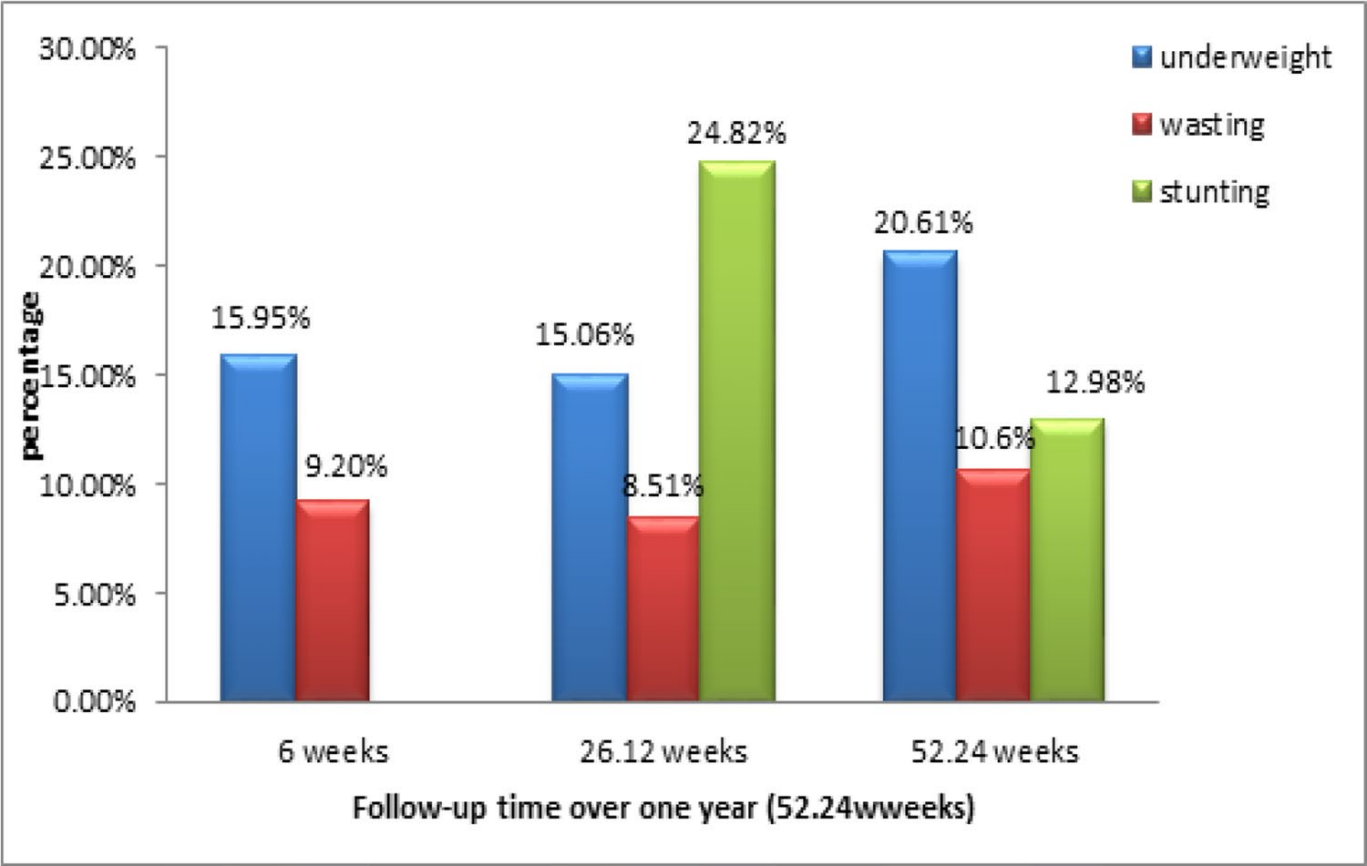
Overall infant nutritional status over 12 months (52.24 weeks) using underweight, wasting and stunting status

The disaggregated nutritional status was as follows: at 6 weeks, 26 (15.95%) infants were underweight, whereas at 6 months (26.12 weeks), stunting was the worst form of malnutrition, with 35 (24.82%) of the infants experiencing stunting. At the end of the study i.e. 12 months (52.24 weeks), 27 (20.61%) infants were underweight infants followed closely by stunting at 17 (12.98%) (Fig. 2).

## Sociodemographic and Socioeconomic Characteristics of the Mothers Living With HIV

A total of 166 mother‒infant pairs were recruited into the study, with 163 (98.2%) mother-infant pairs participating at the beginning of the study (6 weeks). Only 3 (1.8%) mother-infant pairs were censored from the study before 6 weeks of age as follows: at two and a half weeks, one infant transferred from Mathare North Health Center, and two infants died (at 5 weeks and another at 6 weeks). At six months, 22 (13%) mother–infant pairs were Lost To Follow-Up (LTFU), and 10 (6%) more mothers were LTFU at 12 months. The median age of the mothers was 29 years (IQR: 25–33), with 93 (56.0%) of them having an education level of primary level or below. Households mostly consisted of 2–5 members: 121 (72.9%) at recruitment and 88 (67%) at the endpoint. The majority of the mothers were married (141 (84.9%) at recruitment and 113 (86.3%) at the endpoint). Most of the mothers had informal employment: 108 (65.1%) at recruitment and 79 (60.3%) at the endpoint. The majority of the mothers 95 (57.2%) had a household income that ranged between Ksh. 6001–12000 (Table 1).

**Table 1:** Sociodemographic and socioeconomic characteristics of mothers living with HIV

Maternal characteristics	Time-points Recruitment (6 weeks)	Endpoint (12 months or 52.24 weeks)
	Frequency (%) (n = 163)	Frequency (%) (n = 131)
Age in completed years		
18–24	34(20.5)	27(20.6)
25–34	75(45.2)	58(44.3)
35–44	57(34.3)	46(35.1)
Highest Level of Education		
≤ Primary	93(56.0)	75(57.3)
≥ Secondary	73(44.0)	56(42.8)
No. of persons living in the household		
2–5	121(72.9)	88(67.2)
6–8	45(27.1)	43(32.8)
Respondent marital status		
Single	25(15.1)	18(12.7)
Married	141(84.9)	113(86.3)
Employment status		
Formal	27(16.3)	21(16.0)
Informal	108(65.1)	79(60.3)
Unemployed	31(18.6)	31(23.7)
Monthly income (Ksh.)		
≤ 6000	34(20.5)	37(28.2)
6001–12000	95(57.2)	67(51.2)
≥ 12,001–18000	37(22.3)	27(20.6)

## Incidence Rate of HIV Infection among Infants Over a One-Year Follow-up Period

The incidence rate of HIV infection among infants over the one-year follow-up period was 9 cases per 100 person-years (95% CI: 5.465, 16.290).

## Association of Infant Nutritional Status by HIV Outcomes at, 6 Weeks, 6 Months and 12 Months

The relationship between infant nutritional status indicators (underweight, wasting, and stunting) and HIV outcomes was analysed. The results revealed that underweight status was significantly associated with infant HIV status at 6 months (p = 0.009) and 12 months (p = 0.005), but no significant association was found at 6 weeks (p = 1.000). Wasting status was significantly associated with HIV status at all three time points: 6 weeks (p = 0.018), 6 months (p = 0.042), and 12 months (p = 0.005). Stunting status showed significant associations with infant HIV status at 6 months (p = 0.003) and 12 months (p = 0.014). (Table 2).

**Table 2:** Association between infant nutritional outcomes and HIV status at 6 weeks, 6 months, & 12 months

	Recruitment (n = 163)			Time-points 6 months (n = 139)			12 months (n = 131)		
Infant nutritional outcomes	HIV-ve n (%)	HIV + ve n (%)	p-value	HIV-ve n (%)	HIV + ve n (%)	p-value	HIV-ve n (%)	HIV + ve n (%)	P-value
Underweight status			1.000			**0.009**			**0.005**
Normal	131(95.62)	6(4.38)		122(95.73)	5(4.27)		98(94.23)	6(5.77)	
Underweight	25(96.15)	1(3.85)		17(77.27)	5(22.73)		20(74.07)	7(25.93)	
Wasting status			**0.018**			**0.042**			**0.005**
Normal	144(97.3)	4(2.7)		120(94.49)	7(5.51)		109(93.16)	8(6.84)	
Wasted	12(80.0)	3(20.0)		9(75.0)	3(25.0)		9(64.29)	5(35.71)	
Stunting status						**0.003**			**0.014**
Normal				101(97.12)	3(2.88)		106(92.98)	8(7.02)	
Stunted				28 (80.0)	7(20.0)		12(70.59)	5(29.41)	

Bold values indicate statistical significance

## Assessment of Infant Nutritional Status by HIV Outcomes at Recruitment, 6 Month, and 12-Month Follow-ups

Infants who were wasted experienced a higher risk of infant HIV infection across all the three timepoints while infants who were stunted and underweight experienced higher risk of HIV infection only at 6 and 12 months timepoints (Table 3).

**Table 3:** Multivariate analysis between infant nutritional outcomes and HIV status at 6 weeks, 6 months, & 12 months

Infant nutritional status	Relative risk (Rr) of infant HIV infection (95% CI)		
	6 weeks (n = 163)	6 months (n = 139)	12 months (n = 131)
Wasting status			
Normal*(Ref)*	1.00	1.00	1.00
Wasted	**7.40** (1.82, 30.13)	**4.54** **(**1.34, 15.37)	**5.22** (1.97, 13.83)
Underweight status			
Normal*(Ref)*	1.00	1.00	1.00
Underweight	0.88 (0.11, 7.04)	**5.31** (1.67, 16.91)	**4.49** (1.63, 12.32)
Stunting status			
Normal*(Ref)*		1.00	1.00
Stunted		**6.93** (1.89, 25.49)	**4.19** (1.54, 11.37)

Bold values indicate statistical significance

## Child Feeding Practices in the First 6 Months of Life

**Fig. 3:**
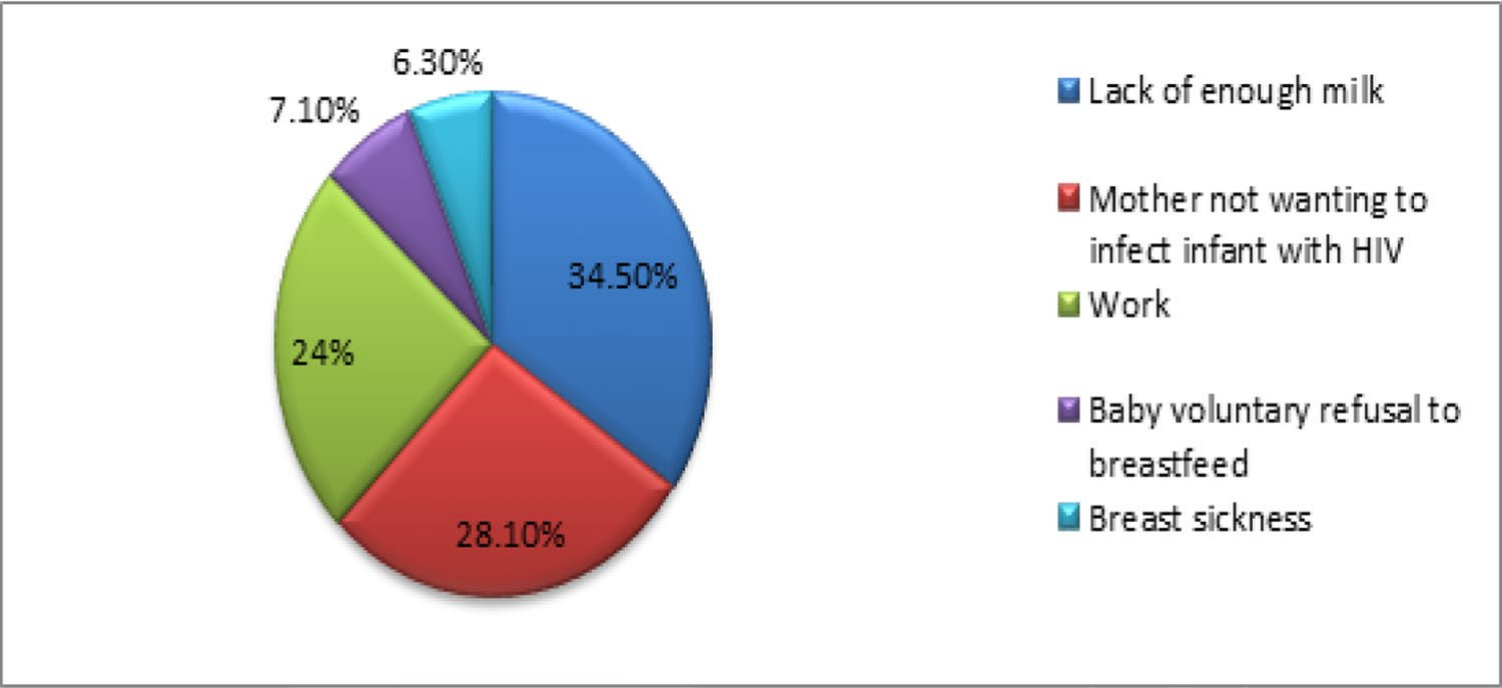
Mothers’ reasons for not exclusively breastfeeding for 6 months

At recruitment (6 weeks), 161 (97.0%) mothers were exclusively breastfeeding (EBF) their infants, 3 (1.8%) did mixed feeding, and 2 (1.2%) were on formula milk. Only 75 (53.2%) of the infants were exclusively breastfed for six months, whereas 64 (45.4%) were mixed fed, and 2 (1.4%) were on formula milk. The mothers cited various reasons for not EBF their infants in the first six months, with the top two reasons being lack of enough breast milk 48 (34.5%) and fear of not wanting to infect their infants with HIV 40 (28.1%) (Fig. 3).

## Maternal Knowledge of Infant Feeding Practices

To determine the mothers’ knowledge of infant feeding practices, mothers were asked questions regarding their knowledge of the available feeding options, if they had been educated on the available feeding options, their source of information on feeding practices, and the feeding option the mother would settle for in the first six months of the infant’s life. Maternal knowledge of feeding practices at recruitment (6 weeks) was very high, with 163 (98.9%) mothers aware that they should exclusively breastfeed their infants for 6 months, 164 (98.2%) mothers choosing EBF, and 2 (1.2%) choosing formula feeding. Most of the 161 mothers (97%) received information on feeding options from antenatal (ANC) clinics.

## Maternal Factors Associated With Infant Nutritional Status at Recruitment (6 Weeks)

Maternal factors associated with infant nutritional status (underweight and wasting status) were assessed at recruitment (6 weeks). Maternal factors significantly associated with infant wasting were maternal age (p = 0.005), maternal employment status (p = 0.002), household income (p = 0.006), ART adherence (p = 0.024), and maternal viral load (0.001). Similar maternal factors (except for maternal age) to those seen in infant wasting status were also seen to be significantly associated with infant underweight status: maternal employment status (p = 0.004), household income (p = 0.010), ART adherence (p = 0.013), and maternal viral load (0.001).

Other maternal factors that were assessed and were not statistically associated with infant wasting status were maternal level of education (p = 0.100), number of persons living in the household (p = 0.761), marital status (p = 0.241), disclosure of HIV status to partners (p = 0.100), knowledge of the mother’s partner(s) HIV status (p = 0.101), maternal HIV staging (p = 0.147), ART regimen (p = 0.067), and maternal Body Mass Index (BMI) (p = 0.059). Maternal factors that were not significantly associated with infant underweight status were similar to those observed in wasting status (except for maternal age) and included: maternal age (p = 0.591), level of education (p = 0.389), number of persons living in the household (p = 0.057), marital status (p = 0.545), disclosure of HIV status to partners (p = 0.100), knowledge of the mother’s partner(s) HIV status (p = 0.101), maternal HIV staging (p = 0.785), ART regimen (p = 0.077), and maternal BMI (p = 0.268).

## Maternal Factors Associated With Infant Nutritional Status at 6 Months (Mid Study)

The maternal factors associated with infant nutritional status (wasting, underweight and stunting) were assessed at 6 months. Maternal factors significantly associated with infant wasting status were maternal age (p = 0.008), maternal employment status (p =  ≤ 0.001), household income (p = 0.001), ART adherence (p = 0.015), maternal viral load (0.002) and maternal BMI (p = 0.003). Other maternal factors assessed and found not to be significantly associated with infant wasting status were level of education (p = 0.770), number of persons living in the household (p = 0.509), marital status (p = 0.400), disclosure of HIV status to partners (p = 0.202), knowledge of the mother’s partner(s) HIV status (p = 0.403), maternal HIV staging (p = 0.479), and ART regimen (p = 0.311).

Maternal factors significantly associated with infant stunting status were maternal age (p = 0.039), maternal employment status (p = 0.012), household income (p = 0.014), ART adherence (p = 0.027), ART Regimen (p = 0.004), maternal viral load (0.003) and maternal BMI (p = 0.011). Other maternal factors assessed and found not to be significantly associated with infant stunting status were level of education (p = 0.171), number of persons living in the household (p = 0.518), marital status (p = 0.791), disclosure of HIV status to partners (p = 0.501), knowledge of the mother’s partner(s) HIV status (p = 0.489), and maternal HIV staging (p = 0.109).

Maternal factors significantly associated with infant underweight status were maternal employment status (p =  ≤ 0.001), household income (p = 0.001), ART adherence (p = 0.036), ART Regimen (p = 0.007) and maternal viral load (0.009). Other maternal factors assessed and found not to be significantly associated with infant stunting status were maternal age (p = 0.412), level of education (p = 0.101), number of persons living in the household (p = 0.310), marital status (p = 0.100), disclosure of HIV status to partners (p = 0.368), knowledge of the mother’s partner(s) HIV status (p = 0.501), and maternal HIV staging (p = 0.170) and maternal BMI (p = 0.324).

## Maternal Factors Associated With Infant Nutritional Status at 12 Months (End-Study)

The maternal factors associated with infant nutritional status (wasting, underweight and stunting) were assessed at 12 months. Maternal factors significantly associated with infant wasting status were maternal age (p = 0.003), maternal employment status (p =  ≤ 0.001), household income (p =  ≤ 0.001), ART adherence (p = 0.020), maternal viral load (0.019) and maternal BMI (p = 0.008). Other maternal factors assessed and found not to be significantly associated with infant wasting status were level of education (p = 0.580), number of persons living in the household (p = 0.100), marital status (p = 0.103), disclosure of HIV status to partners (p = 0.204), knowledge of the mother’s partner(s) HIV status (p = 0.354), maternal HIV staging (p = 0.199), and ART regimen (p = 0.631).

Maternal factors significantly associated with infant stunting status were maternal age (p = 0.030), maternal employment status (p =  ≤ 0.001), household income (p = 0.002), ART adherence (p = 0.019), ART Regimen (p = 0.014) and maternal viral load (p = 0.012). Other maternal factors assessed and found not to be significantly associated with infant stunting status were level of education (p = 0.604), number of persons living in the household (p = 0.100), marital status (p = 0.252), disclosure of HIV status to partners (p = 0.100), knowledge of the mother’s partner(s) HIV status (p = 0.263), maternal HIV staging (p = 0.774) and maternal BMI (p = 0.333).

Maternal factors significantly associated with infant underweight status were maternal employment status (p = 0007), household income (p = 0.013), ART adherence (p = 0.026), ART Regimen (p = 0.027) maternal viral load (p = 0.032) and maternal BMI (p = 0.015). Other maternal factors assessed and found not to be significantly associated with infant stunting status were maternal age (p = 0.611), level of education (p = 0.286), number of persons living in the household (p = 0.100), marital status (p = 1.000), disclosure of HIV status to partners (p = 0.421), knowledge of the mother’s partner(s) HIV status (p = 0.254), and maternal HIV staging (p = 0.222).

## Maternal Factors Influencing Nutritional Status at Recruitment (6 Weeks)

Compared to their older counterparts, younger mothers aged 18–24 years had higher odds of infant wasting relative to their older counterparts. Having some form of employment was a protective factor, with lower odds of infant wasting and underweight status. Similarly, higher household income levels of Ksh. ≥ 12,001–18000 presented lower odds of infant wasting and underweight status. Mothers with poor ART adherence and high viral loads were more likely to have wasted and underweight infants (Table 4).

**Table 4:** Maternal factors influencing infant nutritional status at recruitment

Maternal characteristics n = 163	Infant nutritional status	
	Wasting	Underweight
	OR (95% CI)	OR (95% CI)
Maternal Age		
35–44 *(Ref)*	1.00	
18–24	**aOR 4.31** (1.11, 1.83)	
25–44	0.53(0.11, 2.56)	
Employment status		
Unemployed (*Ref)*	1.00	1.00
Formal	0.21(0.40, 1.10)	**0.21**(0.05, 0.85)
Informal	**0.13** (0.04, 0.43)	**0.21**(0.08, 0.54)
Income (Ksh.)		
≤ 6000 *(Ref)*	1.00	1.00
6001–12000	**0.17** (0.05, 0.56)	**0.28**(0.11, 0.72)
≥ 12,001–18000	**0.17** (0.03, 0.88)	**0.17**(0.48, 0.68)
ART Adherence		
Good *(Ref)*	1.00	1.00
Inadequate	2.84(0.72, 11.14)	2.05 (0.79, 5.32)
Poor	**aOR 5.99**(1.29, 27.93)	**5.73** (1.68, 19.55)
Viral load		
Undetectable (VL) *(Ref)*	1.00	1.00
Low VL	**5.75**(1.39,23.77)	1.45 (0.37, 5.66)
High VL	**8.12**(2.22,29.65)	**aOR 6.33** (2.31, 17.36)

Bold values indicate statistical significance

## Maternal Factors Influencing Infant Nutritional Status at 6 Months

Younger mothers aged 18–24 years had higher odds of having wasted and stunted infants than older mothers aged 35–44 years. Maternal employment and higher household income emerged as protective factors, presenting with lower odds of infant wasting, stunting, and underweight.

Poor adherence to ART and a high maternal viral load were linked to higher odds of infant wasting, stunting, and underweight than good ART adherence and undetectable viral loads. Compared with mothers on first-line regimens, mothers on second-line ART regimens had higher odds of having stunted and underweight infants. Finally, underweight mothers had greater odds of wasting and stunted infants than mothers with a normal body mass index (BMI); however, maternal BMI did not influence infants’ stunting or underweight status at 6 months (Table 5).

**Table 5:** Maternal factors influencing infant nutritional status at 6 months

Maternal characteristics	Infant nutritional status		
	Wasting	Stunting	Underweight
n = 141	OR (95%CI)	OR (95%CI)	OR (95%CI)
Maternal Age			
35–44 *(Ref)*	1.00	1.00	
18–24	**aOR = 5.46** (1.09, 27.34)	**3.40** (1.22, 9.48)	
25–34	0.51 (0.08, 3.35)	1.23 (0.48, 3.19)	
Employment			
Unemployed (*Ref)*	1.00	1.00	1.00
Formal	0.45 (0.07, 3.00)	0.58 (0.18, 1.90)	0.43 (0.12, 1.51)
Informal	**aOR = 0.05** (0.01,0.26)	**0.26** (0.10, 0.67)	**aOR = 0.10** (0.03, 0.32)
Income (Ksh.)			
≤ 6000 *(Ref)*	1.00	1.00	1.00
6001–12000	**0.08** (0.01, 0.38)	**0.26** (0.11, 0.65)	**0.13** (0.04, 0.39)
≥ 12,000–18000	**0.20** (0.34, 1.03)	0.35 (0.12, 1.07)	0.24 (0.07, 0.84)
ART Adherence			
Good *(Ref)*	1.00	1.00	1.00
Inadequate	2.03 (0.48, 8.56)	1.62 (0.68, 3.85)	1.88 (0.67, 5.30)
Poor	**8.78** (1.86, 41.5)	**4.89** (1.44,16.61)	**5.14** (1.37,19.22)
ART Regimen			
First-line *(Ref)*		1.00	1.00
Second-line		**aOR = 5.49** (1.64,18.38)	**5.89** (1.75,19.85)
Viral Load (VL)			
Undetectable (*Ref)*	1.00	1.00	1.00
Low VL	1.00 (0.11, 9.48)	0.87 (0.26, 2.87)	1.23 (0.31, 4.91)
High VL	**9.06** (2.38,34.55)	**4.86** (1.87,12.66)	**4.92** (1.71,14.19)
Mother BMI			
Normal *(Ref)*	1.00	1.00	
Underweight	**13.75** (2.74, 69.08)	**aOR = 4.76** (1.36,16.65)	
Overweight	1.13 (0.18, 7.08)	0.55 (0.19, 1.55)	
Obese	3.14 (0.48, 20.73)	0.72 (0.16, 3.22)	

Bold values indicate statistical significance

## Maternal Factors Influencing Infant Nutritional Status at 12 Months

Younger mothers, aged 18–24 years, had higher odds of infant wasting relative to their older counterparts, however, maternal age did not influence infant stunting or underweight status. Maternal employment and having higher household income emerged as protective factors, presenting with lower odds of infant wasting, stunting, and underweight status.

Mothers with poor ART adherence and high viral loads had greater odds of infant wasting, stunting, and underweight than mothers with good adherence and undetectable viral loads. Mothers on second-line ART regimens were more likely to experience higher odds of infant stunting and underweight status than were those on first-line regimens. Underweight mothers had greater odds of infant wasting than did those with a normal BMI; however, maternal BMI did not influence infant stunting or underweight status at 12 months (Table 6).

**Table 6:** Maternal factors influencing infant nutritional status at 12 months

Maternal characteristics	Infant nutritional status		
	Wasting	Stunting	Underweight
n = 131	OR (95%CI)	OR (95%CI)	OR (95%CI)
Maternal Age			
35–44 *(Ref)*	1.00	1.00	
18–24	**aOR = 6.83** (1.41,32.93)	1.43 (0.43, 4.70)	
25–34	0.84 (0.15,4.74)	0.25 (0.06,1.02)	
Employment			
Unemployed (*Ref)*	1.00	1.00	1.00
Formal	**0.11** (0.01, 0.95)	**0.10** (0.01, 0.82)	0.24 (0.06, 1.02)
Informal	**0.08** (0.02,0.33)	**0.12** (0.04,0.40)	**aOR = 0.22** (0.09, 0.59)
Income (Ksh.)			
≤ 6000 *(Ref)*	1.00	1.00	1.00
6001–12000	**0.07** (0.02,0.36)	**0.19** (0.06, 0.62)	0.29 (0.11,0.76)
≥ 12,001–18000	**0.09** (0.01, 0.76)	**0.09** (0.01, 0.76)	**0.20** (0.05, 0.81)
ART Adherence			
Good *(Ref)*	1.00	1.00	1.00
Inadequate	2.77 (0.68,11.24)	1.21 (0.32, 4.64)	0.77 (0.27, 2.20)
Poor	**aOR = 7.03** (1.29,38.24)	**4.11** (1.14,14.82)	**3.97** (1.28, 12.3)
ART Regimen			
First-line *(Ref)*		1.00	1.00
Second-line		**3.68** (1.09,12.49)	**3.96** (1.20,13.05)
Viral Load (VL)			
Undetectable *(Ref)*	1.00	1.00	1.00
Low VL	3.58 (0.76,16.68)	1.54 (0.29, 8.21)	1.78 (0.50, 6.33)
High VL	**4.98** (1.37,18.06)	**5.14** (1.65,16.08)	**3.61** (1.34, 9.70)
Mother BMI			
Normal *(Ref)*	1.00	1.00	
Underweight	**8.06** (1.87,34.68)	2.29 (0.51,10.36)	
Overweight	0.51 (0.10, 2.67)	0.50 (0.13, 1.96)	
Obese	0.64 (0.07, 5.81)	0.87 (0.17, 4.53)	

Bold values indicate statistical significance

## Final Models for Maternal Factors Influencing Infant Nutritional Status at 6 Weeks, 6 Months and 12 Months via the Bayesian Information Criterion (BIC)

Modelling was conducted via postestimation techniques, applying the Bayesian information criterion (BIC) to select the best model among several competing models (utilizing both backwards and forward stepwise methods). In the final infant wasting model, younger maternal age (18–24 years) was associated with higher odds of infant wasting across all three time points: at 6 weeks aOR 4.31 (CI:1.11, 1.83), at 6 months aOR 4.49 (CI:1.09, 27.34), and at 12 months aOR 5.49 (CI: 1.41, 32.97). Mothers who were employed had lower odds of infant wasting OR 0.05 (CI: 0.01, 0.26) at 6 months. However, at 12 months mothers with poor ART adherence experienced higher odds of infant wasting aOR 7.03 (CI: 1.29, 38.24) (Table 7).

**Table 7:** Final model for maternal factors influencing infant wasting at various time points

Maternal characteristics n = 139	aOR	Robust Std. Err	z	P > z	[95% CI]		Prob > Chi2
					Lower	Upper	
*Final model for wasting at recruitment*							
Maternal Age							**0.015**
35–44 *(Ref)*	1.00						
18–24	4.31	2.99	2.11	**0.035**	1.11	1.83	
25–34	0.53	0.42	− 0.80	0.426	0.11	2.56	
ART Adherence							
Good *(Ref)*	1.000						
Inadequate	2.84	1.98	1.49	0.136	0.72	11.14	
Poor	5.99	4.70	2.28	**0.023**	1.29	27.93	
*Final model for wasting at 6 months*							
Maternal Age							** < 0.001**
35–44 *(Ref)*	1.00						
18–24	5.46	4.49	2.07	**0.039**	1.09	27.34	
25–34	0.51	0.49	0.70	0.487	0.08	3.35	
Employment status							
Unemployed (*Ref)*	1.00						
Formal	0.45	0.43	− 0.83	0.406	0.07	3.00	
Informal	0.05	0.04	− 3.59	** < 0.001**	0.01	0.26	
*Final model for wasting at 12 months*							
Maternal Age							**0.002**
35–44 *(Ref)*	1.00						
18–24	6.83	5.49	2.39	**0.017**	1.41	32.973	
25–34	0.84	0.74	− 0.20	0.843	0.15	4.739	
ART Adherence							
Good *(Ref)*	1.00						
Inadequate	2.77	1.98	1.43	0.153	0.68	11.24	
Poor	7.03	6.07	2.26	**0.024**	1.29	38.24	
*Summary for wasting models*							
Timepoint	n	Log-Likelihood	AIC	BIC	Persons GOF *X *^*2*^	GOF p value	DF
Recruitment	n = 163	− 42.13	94.26	109.74	6.98	0.137	5
6 months	n = 139	− 28.36	66.73	81.41	1.98	0.74	5
12 months	n = 129	− 35.75	81.51	95.81	4.45	0.35	5

Bold values indicate statistical significance

Modelling for stunting at 6 months revealed that underweight mothers and those on second-line ART regimens had higher odds of infant stunting, with aOR 4.67 (CI: 1.36, 16.65) and aOR 5.49 (CI: 1.64, 18.38) respectively. At 12 months, mothers with poor ART adherence and those on second-line ART regimens also presented increased odds of infant stunting, with aOR 4.11 (CI: 1.14, 14.81) and aOR 3.68 (CI: 1.09, 12.49) respectively (Table 8).

**Table 8:** Final model for maternal factors influencing infant stunting at various time points

Maternal characteristics	aOR	Robust Std. Err	z	P > z	[95% CI]		Prob > Chi2
					Lower	Upper	
*Final model for stunting at 6 months*							
ART Regimen							0.002
First-line *(Ref)*	1.00						
Second-line	5.49	3.37	2.76	**0.006**	1.64	18.379	
*Mother BMI*							
Normal *(Ref)*	1.00						
Underweight	4.76	3.04	2.44	**0.015**	1.36	16.65	
Overweight	0.55	0.29	-1.14	0.256	0.19	1.55	
Obese	0.72	0.55	-0.43	0.665	0.16	3.22	
*Final model for stunting at 12 months*							
ART Adherence							0.010
Good *(Ref)*	1.00						
Inadequate	1.21	0.83	0.28	0.782	0.32	4.639	
Poor	4.11	2.69	2.16	**0.031**	1.14	14.815	
ART Regimen							
First-line *(Ref)*	1.00						
Second-line	3.68	2.30	2.09	**0.036**	1.09	12.49	
*Summary for stunting models*							
Timepoint	n	Log-Likelihood	AIC	BIC	Persons GOF ***X ***^***2***^	GOF **p value**	DF
6 months	n = 139	-69.39	148.78	163.45	1.56	0.669	3
12 months	n = 129	-45.21	98.42	109.86	2.88	0.236	2

Bold values indicate statistical significance

In the final underweight model, a high maternal viral load aOR 6.33 (CI: 2.31, 17.36) presented higher odds of infant underweight status, whereas employed mothers had lower odds of infant underweight status at both 6 and 12 months aOR 0.10 (CI: 0.03, 0.32) and aOR of 0.22 (CI: 0.09, 0.59) respectively (Table 9).

**Table 9:** Final model for maternal factors influencing infant underweight status at various time points

Maternal characteristics	aOR	Robust Std. Err	z	P > z	[95% CI]		Prob > Chi2
					Lower	Upper	
*Final model for Underweight at recruitment*							
Viral Load (VL)							0.002
Undetectable (VL) *(Ref)*	1.00						
Low VL	1.45	1.01	0.54	0.591	0.37	5.66	
High VL	6.33	3.26	3.59	** < 0.001**	2.31	17.36	
*Final model for Underweight at 6 months*							
Employment status							< 0.001
Unemployed (*Ref)*	1.00						
Formal	0.43	0.28	− 1.32	0.188	0.12	1.51	
Informal	0.10	0.06	− 3.96	** < 0.001**	0.03	0.32	
*Final model for Underweight at 12 months*							
Employment status							0.007
Unemployed (*Ref)*	1.00						
Formal	0.24	0.18	− 1.94	0.053	0.06	1.02	
Informal	0.22	0.11	− 3.05	**0.002**	0.09	0.59	
*Summary for underweight models*							
Timepoint	n	Log-Likelihood	AIC	BIC	DF		
Recruitment	n = 163	12.98	136.72	146.00	3		
6 months	n = 141	15.80	110.441	119.29	3		
12 months	n = 130	10.06	128.84	137.44	3		

Bold values indicate statistical significance

## Discussion

### Maternal Factors Influencing Infant Nutritional Status

Globally, 2.7 million (45%) deaths in children are attributed to undernutrition annually [14]. Optimal nutrition in a child’s first 2 years of life is crucial for ensuring a reduction in mortality, morbidity, and risk of chronic diseases and ensuring good development. Evidence has demonstrated that HIV-exposed uninfected infants have worse nutritional status than HIV-unexposed infants even when their mothers are receiving life-long ART [15]. Exposure to HIV and ART in utero (especially protease inhibitor-based regimens) has been linked to increased low birth weight and preterm birth, which are universal risk factors for increased morbidity and mortality among these infants [16].

Exclusive breastfeeding (EBF) in infancy for 6 months has many benefits, such as the prevention of malnutrition, gastroenteritis and other diseases that increase infant mortality and improve development, especially cognitive development [14, 17, 18]. EBF was performed in 161 (97.0%) of the infants at 6 weeks but rapidly declined over six months, whereas only 75 (53.2%) of the infants were exclusively breastfed up to six months of life. EBF was much lower in Cameroon, where 243 (89%) infants breastfed for the first five months of life [19]. However, in a West Cape study, no infant was exclusively breastfeeding at 6 months [17]. Despite the majority of the mothers (163, 98.9%) knowing that they should EBF their infants for the first 6 months, with similar findings in Nigeria (95.3%) [20], mothers still mixed and fed their infants before six months were over. They cited various reasons for mixed feeding, with the top three reasons being lack of enough breast milk, fear of not wanting to infect their infants with HIV and work commitments. Similarly, “return to work” was cited as the most frequent reason for not EBF for six months [21] and “perceived insufficient breast milk by the mother” [22]. Mixed feeding before six months of life increases the risk of infant HIV positivity [23], which implies that these infants are most likely not able to obtain the benefits of exclusive breastfeeding for six months.

The maternal socio-demographic factors that influence infant nutritional status are maternal age, employment status, and household income. Compared with their older counterparts, younger mothers aged 18–24 years presented greater odds of wasting and stunting across all the three time points i.e. at 6 weeks, 6 months and 12 months (Tables 4, 5 and 5). Younger mothers < 25 years of age were less likely to practice EBF at 0–5 months aOR 0.19; (95%CI 0.41, 0.85; p = 0.030) than their older counterparts aged 26–34 years were more likely to practice EBF exclusively [24]. This increased the risk of malnutrition among their infants. This finding differed from that in Tanzania [25] whereby maternal age was not a prognostic factor for infant nutritional status.

Unemployed mothers with low household income levels experienced more infant stunting, underweight, and wasting in the current study. Similar findings were reported in resource limited settings [26] whereby mothers with low socioeconomic status experienced more stunting in their infants due to inadequate intake of the required nutrients. Similarly, in Tanzania [25], infants from lower socio-economic backgrounds experienced more infant underweight and wasting nutritional status, which resulted in impaired infant growth in these resource-limited households. Maternal education levels and marital status were not associated with infant nutritional status, with similar findings by [15, 20] but differed from that of [8], who reported that higher levels of maternal educational attainment were associated with a decreased risk of stunting (p < 0.01) in the infant’s first two years of life.

Poor maternal ART adherence led to poor nutritional outcomes (stunting, underweight, and wasting) relative to mothers with good ART adherence, a finding similar to that of a study conducted in Malawi [5]. Poor ART adherence increases the risk of maternal treatment failure, poor health and increased risk of vertical transmission of HIV [27].

Across all time points, mothers with high viral load counts had higher odds of wasting, stunting, and underweight. [8] reported that a high maternal viral load increased the risk of stunting in infants, with similar findings by [28], which revealed that a high maternal viral load (immunosuppression) was associated with infant stunting and underweight. High maternal viral load was often associated with maternal illness and increased risk of maternal opportunistic infections, and subsequent poor infant feeding and growth.

Mothers who were on the second-line regimen of ART were more likely to be stunted in the current study. Mothers switching from the first-line regimen to the second-line regimen are characterized by periods of high viremia during the ART switching period, which is more common than not related to ART non-adherence. Continuous and good ART adherence improves infant nutritional status during pregnancy and the breastfeeding period [29].

Maternal BMI was associated with an increased risk of infant wasting and stunting at 6 months and underweight and wasting at 12 months in the current study, with similar findings [30, 31]. [32] reported that poor nutritional status in HIV-positive mothers may impair immunity and weaken epithelial integrity, which is associated with the vertical transmission of HIV. In Mozambique, HIV-positive mothers are put on nutritional supplements during pregnancy and the lactation period, which have been shown to improve infant nutritional outcomes, maternal retention in the Prevention of Mother To Child Transmission (PMTCT) of HIV programs, the reduction in premature deliveries, and the reduction in infant low birth weight [28].

## Conclusion

Inadequate maternal nutritional status presented increased odds of infant stunting which is the most severe form of malnutrition in infants. Consequently, stunted infants were at a higher risk of HIV transmission. Additionally, poor maternal ART adherence and high maternal viral load levels also presented increased odds of poor infant nutritional status. We recommend the integration of the full nutritional package schedule into the PMTCT and EID policies to facilitate early screening and management of malnutrition in pregnant and breastfeeding women and their infants, which will decrease the risk of vertical transmission of HIV and subsequent poor infant growth. This will not only promote adherence to recommended infant feeding practices but also encourage mothers living with HIV to make informed nutritional choices for themselves and for their infants. Further, enhanced ART adherence monitoring for young mothers should be implemented to improve ART adherence and subsequently overall wellbeing of the mothers, and reduced risk of infant HIV transmission during pregnancy, birth and the breastfeeding period.

## Limitations of the Study

The nutrition data were collected at specific time points (6 weeks, 6 months, and 12 months), making it difficult to capture the rapid fluctuations in the wasting and underweight nutrition parameters that can occur over a given year. However, these parameters were taken over one year and were able to show trends and changes over time that could be useful in decision-making.

The study sample consisted of mothers attending the EID clinic within the catchment area of informal and low-income settlements of Nairobi, which may not have been representative of the general population of mothers living with HIV with infants aged 0–12 months. These findings could, therefore, be generalized only to studies conducted in similar circumstances.

Positionality: Whilst this was a quantitative research focusing on objectivity and reproducibility, as researchers, we recognize that our identities and roles inherently influenced our interactions with participants and interpretation of their responses. Our research team comprised of individuals with diverse backgrounds in public health, community development, and socio-cultural integration, with prior experience working within the studied community. This familiarity facilitated trust and open discussions during the administration of semi-structured questionnaires but also required vigilance against potential biases stemming from preconceived notions.

We acknowledge that our professional roles might have positioned us as authorities, potentially influencing participants’ responses. To address this, we emphasized our role as facilitators, fostering mutual respect, and ensuring participants’ perspectives were central to the study’s outcomes.

Reflexivity was integral to our process, involving team discussions, journaling, and critical evaluation of assumptions. These practices minimized undue influence on the data while acknowledging the inevitable impact of our positionality, enhancing the study’s transparency and credibility.

## Data Availability

The minimal underlying data set can be provided as part of supporting files for reviewer access. The data set will be de-identified, however, the data set remains part of the Government of Kenya, Ministry of Health property, and permission to share it publicly needs to be sought from the director general using the following contacts: Dr. Patrick Amoth Ag. Director General for Health at Ministry of Health Ministry of Health, Afya House, Cathedral Road, P.O. Box: 30016–00100, Nairobi, Kenya. Email: dghealth2019@gmail.com.

## Abbreviations

HIV: Human immunodeficiency virus
EID: Early infant diagnosis
ART: Antiretroviral therapy
PMTCT: Prevention of mother to child infection
BMI: Body mass index
EBF: Exclusive breastfeeding
PCR: Polymerase chain reaction
